# Cardiac angiosarcoma: A case report and review of current treatment

**DOI:** 10.1097/MD.0000000000018193

**Published:** 2019-12-10

**Authors:** Qian Linfeng, Xu Xingjie, Davies Henry, Wan Zhedong, Xu Hongfei, Zhao Haige

**Affiliations:** Department of Cardiothoracic Surgery, The First Affiliated Hospital of Zhejiang University, Hangzhou, China.

**Keywords:** cardiac angiosarcoma, chemotherapy, palliative surgery, radiotherapy

## Abstract

**Rationale:**

Primary cardiac angiosarcoma is a rare malignant cardiac neoplasm with early metastasis and poor prognosis. As there are currently no guidelines or effective therapeutic strategies, management of this condition depends on previous experiences of the clinician treating and the consideration of reported cases.

**Patient concerns:**

A 65-year-old male presented to our department with a 4-day history of chest tightness, dyspnea, lower extremity weakness and occasional dizziness, and a transthoracic echo (TTE) revealed a right atrium occupying mass.

**Diagnoses:**

TTE showed right atrium occupation, and the post-operative histopathology showed the tumor to be a primary cardiac angiosarcoma.

**Interventions:**

Right atrium tumor resection and right atrium reconstruction with a bovine pericardium were performed.

**Outcomes:**

The patient recovered from surgery and discharged but died 10 months after surgery because of complete resection was impossible and adjuvant therapy was not performed.

**Lessons:**

Resection of primary cardiac angiosarcomas should be integrated with a combination of chemotherapy, radiotherapy, and targeted therapy based on tumor cell gene mutation and altered expression.

## 1 Introduction

Primary cardiac neoplasms are rare malignant tumors, and of these angiosarcoma is the most common histopathology type.^[1]^ Due to the rarity, early blood metastasis and resistance to chemoradiotherapy, prognosis of angiosarcoma patients is predominantly poor. We report a case of a right atrium angiosarcoma treated with palliative surgery alone who unfortunately ended up with brain metastases. Also, a review of current treatment strategies and reported cases revealed that there is currently no truly effective treatment for primary cardiac angiosarcomas, but targeted immunotherapy shows promising potential.

## 2 Case report

The patient was a 65-year-old Chinese male who presented with chest tightness, dyspnea, lower extremity weakness and occasional dizziness lasting for around 4 days. His past medical history was significant for hypertension (1 year), but had been well controlled with medicine and a cholecystectomy 20 years previously (due to acute cholecystitis). In addition, the patient had been on follow-up for a protein positive urine for 4 years. Physical examination was unremarkable except for mild edema of both lower and upper extremities, especially the left hand. Before presentation to our hospital, investigation at the local clinic had pointed to cardiac and pulmonary abnormalities. Computed tomography (CT) had demonstrated a multifocal high-density shadow and lymph node enlargement (including the right upper lobe) with bilateral pleural effusions, a pericardial effusion and an enlargement of the cardiac area. Following this he came to our hospital for further investigation and treatment.

**Figure 1:**
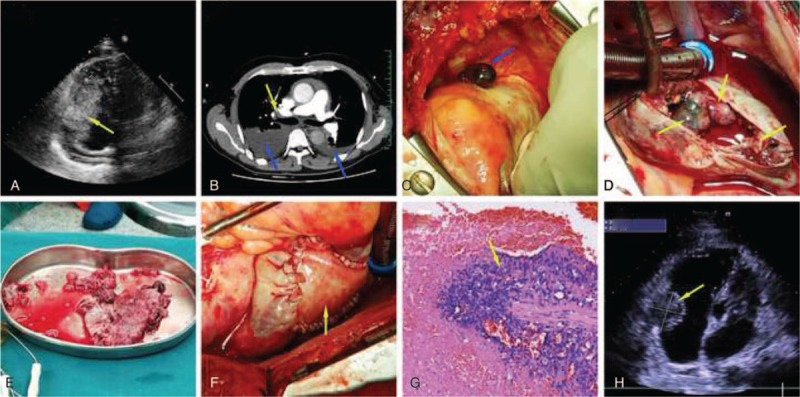
(A) Preoperative transthoracic echocardiography (TTE) revealed an iso-echoic mass (8.0 × 4.8 cm) with a broad base (3.6 cm, from tricuspid inferior leaflet annulus to the mural) in right atrium. The mobile mass entered into the tricuspid orifice in diastole, and back in to the right atrium in systole. (B) Preoperative computed tomography angiography (CTA) demonstrated right superior pulmonary artery embolism (yellow arrow) and pleural effusion (blue arrow). (C) The neoplasm penetrated the right atrium. (D) The right atrium was opened and tumor mass (yellow arrow) attached to the atrial wall were seen. (E) Resected tumor mass. (F) Right atrium was reconstructed by bovine pericardium. (G) Microscopic examination showed apparent hemorrhage and necrosis within, unequally distributed spindle and ovoid cells with obvious atypia and mitotic figures which had invaded and spread locally. A characteristic anastomosing vascular channel line with atypical cells was also observed. (H) Postoperative TEE revealed the remaining hypoechoic mass (3.5 cm × 2.8 cm) was separated from the right atrium chamber by hyperechoic bovine pericardium.

After admission, transthoracic echocardiography (TTE) showed a right atrium occupation (8.0 × 4.8 cm) with a broad base (Fig. 1A), right ventricular enlargement, a widened inferior vena cava, mild tricuspid regurgitation, moderate pericardial effusion, bilateral pleural effusions, tachycardia, aortic sclerosis, and left ventricular diastole deterioration. Preoperative transesophageal echocardiography (TEE) showed the same diagnoses, however it also elucidated the penetration of the mass into the pericardium and several smaller masses attached to the right atrium wall and tricuspid. Pulmonary artery computed tomography angiography (CTA) demonstrated a microembolism in the right upper lobe of the lung (Fig. 1B). A head MRI showed no sign of metastasis to the brain at the time. Blood tests showed hypoxemia, and a slightly elevated blood urea nitrogen, creatinine, uric acid, aspartate aminotransferase, and homocysteine. It also showed an increased direct bilirubin, an international normalized ratio of 1.31, and a gamma-glutamyl transferase of 109 U/L.

During the surgery, a median sternotomy was performed followed by a “T” shape pericardiotomy demonstrating that the tumor had penetrated through the right atrium (Fig. 1C) and caused a large pericardial effusion. Next, the right atrium and right ventricular outlet was opened longitudinally showing the tumor spreading throughout the endocardium of right atrium with abundant tumor tissue between the papillary muscles of the tricuspid valves (Fig. 1D). The tumor was resected as much was possible (Fig. 1E), and the tricuspid valves were then reconstructed. Complete resection of the tumor was not possible as infiltration was extensive. A bovine pericardium was then sutured to cover the unresectable part of the tumor, above the tricuspid isthmus, and to reconstruct the right atrium (Fig. 1F). The right pulmonary artery emboli were removed by suction through the opened pulmonary artery. Finally, pericardial and mediastinum drainage tubes were placed. The patient was then transferred to surgical intensive care unit for close observation. Postoperative TEE showed a small hypoechoic mass remaining (0.6 × 0.5 cm) (Fig. 1H) and mild tricuspid regurgitation.

Histopathologic and immunohistochemical investigation confirmed the diagnosis to be angiosarcoma (high grade) positive for CD34, CD31, F8-R-Ag and Vimentin. Spindle and ovoid cells were present, as was a characteristic anastomosing vascular channel (Fig. 1G). Nevertheless, as CD117 and DOG-1 were both positive, advanced genetic testing was recommended to exclude a gastrointestinal mesenchymal tumor. After surgery the patient underwent supportive treatment only, patient and his family refused to do any adjuvant chemotherapy or radiation therapy. The recovery from surgery was uneventful and the patient was discharged 10 days later from our hospital. Unfortunately, 4 months later the patient was found to have brain metastases, and dead 10 months later, after the surgery.

## 3 Discussion

Primary tumors of the heart are rare, with incidences ranging from 0.001% to 0.030% at autopsy. Among them only one-quarter are malignant.^[1]^ Most of the malignant tumors are sarcomas and around 40% are angiosarcomas.^[2]^ The main complaints in patients with angiosarcomas arise from: tumor mass obstruction, myocardial or atrial wall invasion and penetration which can cause arrhythmias and pericardial effusions, emboli, systemic symptoms like fever or weakness, metastases.

In our patient, extremity edema may have been caused by blood flow obstruction and/or pericardial effusions. Chest tightness, dyspnea, dizziness, and weakness were most likely the result of the pulmonary embolism, plural effusions, and consequent hypoxemia. The common location of metastases includes the lungs, liver, brain, lymph nodes, bones, adrenal glands, and spleen.^[12]^ In the case of our patient, brain metastases were subsequently found and it is possible that the pulmonary nodes were also metastasis location of angiosarcoma. However, they could also have been inflammation, as it was hard to determine by CT imagine.

The diagnosis approach of angiosarcoma is based on TTE, CT, magnetic resonance imaging, histopathology and immunohistochemistry. The most widely utilized techniques of echocardiogram are TEE and TTE. In the most case, TTE is the initial examination for its convenience, and it has a 75% sensitivity for visualizing primary cardiac angiosarcomas.^[8]^ However, TEE should also be considered, as it has superior image resolution and better visualization, which can provide more information about the valves, the site of tumor implantation and wall infiltration, especially those located in the posterior cardiac structure.^[3–8]^ Cardiac magnetic resonance (CMR) and CT can provide more information about tissue characterization, precise location, and myocardial invasion. In addition, CMR is helpful in differentiation of benign and malignant tumors.^[9]^ Before planning the treatment strategy, the malignancy and metastasis should be evaluated. (18)F-FDG positron emission tomography–computed tomography is a noninvasive preoperative test to reveal any potential metastases.^[10]^ Due to unaccepted bleeding risk of biopsy and low sensitivity, pericardial fluid cytology testing is rarely used. A variety of echocardiography and CT findings are used to assess the malignancy of the tumor such as:

(1) wide and poorly defined attachment to the myocardium and/or broad base,
(2) evidence of central necrosis and inhomogeneous,
(3) right chamber occupation,
(4) pericardial effusion,
(5) local invasion,
(6) large or irregular shape.^[2,8]^

However, the definitive diagnosis and gold standard is cytology and immunohistochemistry. In the HE slide, angiosarcoma often shows abnormal mitosis with epithelioid, spindle, plasmacytic shaped cells and multiple prominent or bar-shaped nucleoli and chromatin strands. The characteristic vasoformative features include hemophagocytosis, cytoplasmic lumnia/vacuoles containing red blood cells, and endothelial wrapping.^[11]^ As some other tumors can produce prominent vascular structures in the stroma, immunohistochemical staining is necessary. CD31, CD34, FKI-1, and von Willebrand factor are the most commonly used markers; however, mutation and poor differentiation of tumor cells may cause misdiagnosis, so comprehensive consideration of all these diagnostic approaches is important.^[12]^

Treatment guidelines for cardiac angiosarcoma are not yet established due to the rarity, but multidiscipline therapies including surgery, radiotherapy and chemotherapy are most commonly employed.

Surgery is the mainstay of angiosarcoma treatment. According to the research of Blackmon SH, median overall survival (OS) of cardiac sarcoma is 12 months with 17 months for R0 resection and 6 months for R1 resection. For right heart sarcoma surgery combined with neoadjuvant chemotherapy, the OS is 27 months and longest survival time is 9.5 years. For left sided sarcomas, orthotopic transplantation is employed due to the poor exposure.^[13]^ More recent reports from a single institution study demonstrate neoadjuvant chemotherapy with doxorubicin/ifosfamide before the surgery can improve estimated median survival to 15.5 months for tumors in the right heart chamber and 20 months in the left.^[14]^ Heart transplantation is an alternative method for sarcoma treatment and this with postoperative adjuvant chemotherapy shows no significant difference in OS.^[15,16]^ Therefore, complete resection with neoadjuvant chemotherapy is the preferred method of treatment. When total resection of the tumor is not possible, partial resection can relieve the hemodynamic obstruction and improve short term symptoms. Cardiac sarcoma is not an indication for heart transplantation.

Radiotherapy usage in cardiac sarcoma is difficult as the beat of the heart and respiratory movement both make it difficult to focus the beam, which leads to damage of the surrounding tissue damage and radiation-induced cardiac-toxicity. But the development of respiratory gating, breath-hold techniques, tumor tracing, intensity-modulated radiotherapy and four-dimensional tumor motion analysis provide some opportunity to accurately target mobile tissue during radiotherapy.^[17]^ In a case of a patient critically ill from having an angiosarcoma, chemoradiotherapy with an external radiotherapy beam to a dose of 50 Gy in 25 fractions with paclitaxel was employed weekly, the symptoms were relieved and tumor burden was decreased.^[18]^

Most cardiac angiosarcomas are found in the late stage with distal metastasis and local invasion making complete resection impossible, thus chemotherapy is also important. Due to the rarity of cardiac angiosarcomas, most treatment modality available is on extra-cardiac angiosarcomas.^[17]^ The mainstay of cytotoxic chemotherapy drugs for angiosarcomas is anthracycline, ifosfamide and taxanes (paclitaxel and docetaxel).^[19]^ A clinical trial of first-line anthracycline-based chemotherapy for angiosarcoma demonstrated a median progression free survival of 4.8 months and an overall survival 9.9 months, which is not significantly different from other soft tissue sarcomas.^[20]^ Another study showed that ifosfamide combined with either doxorubicin or docetaxel both have the same response but better overall survival, 17 months.^[21]^ Single-agent doxorubicin used once as first-line therapy for angiosarcoma, but a study by ltaliano A showed that weekly paclitaxel seemed to have similar efficacy for metastatic angiosarcoma.^[22]^

Commonly used targeted drug therapy focuses on vascular endothelial growth factor A and tyrosine kinase, and includes imatinib, sorafenib, pazopanib, and bevacizumab. In the clinical trials, imatinib, sorafenib, and bevacizumab show limited activation against angiosarcomas, but increase toxicity, especially when combined with paclitaxel. Pazopanib activity in angiosarcomas is comparable to other soft tissue sarcomas, with a progression-free survival 3 months and an overall survival of 9.9 months.^[23–26]^ However, there has also been success in treating some cases of cardiac angiosarcoma reported. A combination of docetaxel and radiotherapy kept 2 patients progression free for 12 months and 16 months, respectively.^[27,28]^ Postoperative doxorubicin and ifosfamide resulted in a patient surviving for in excess of 96 months.^[29]^ In another case, a primary metastatic cardiac angiosarcoma treated with taxanes and pazopanib lead to remission of pulmonary and liver metastasis.^[30]^

Our patient had resection of the large bulk of his angiosarcoma, from which the recovery was smooth, yet he still died from the tumor metastasizing to the brain. Since the patient died of brain metastasis, the pulmonary mass can be a tumor emboli. However incomplete resection with no adjuvant therapy also could be a reason for metastasis to the brain. This underlies the aggressiveness of cardiac angiosarcomas and the importance of treating them concomitantly with adjuvant chemo or radiotherapy.

Further randomized controlled clinical trials are needed to standardize and improve the treatment of this devastating condition. Research into angiosarcoma mutations and corresponding target drugs may also offer some alternative therapeutic pathways.

## Author contributions

**Conceptualization:** Xu Hongfei.

**Data curation:** Davies Henry, Wan Zhedong.

**Funding acquisition:** Xu Hongfei.

**Project administration:** Haige Zhao.

**Software:** Wan Zhedong.

**Visualization:** Xu Xingjie.

**Writing – original draft:** Qian Linfeng, Davies Henry.

**Writing – review & editing:** Haige Zhao.
